# Brain activity during acquisition of long visuospatial sequences

**DOI:** 10.3389/fcogn.2025.1493709

**Published:** 2025-09-01

**Authors:** Milena I. Mihovilovic, Thomas Stephan, Andreas Straube, Marianne Dieterich, Thomas Eggert

**Affiliations:** ^1^Department of Neurology, LMU University Hospital, Munich, Germany; ^2^German Center for Vertigo and Balance Disorders-DSGZ, LMU University Hospital, Munich, Germany

**Keywords:** explicit memory, fMRI, sequence learning, visuospatial processing, spatial attention, superior parietal lobule, V5, middle frontal gyrus

## Abstract

Explicitly acquiring a visuospatial sequence involves various fundamental attentional and processing mechanisms that can be difficult to disentangle. To this end, we performed an fMRI study (*n* = 34) on the acquisition of visuospatial targets in a delayed imitation paradigm. Task phases alternated between presentation and recall of a 20-target-long sequence. Behavioral data from the recall phase was used to determine encoding progress as a function of time during presentation, with this progress taken as a continuous predictor of BOLD activity. A separate, attention-only task was devised in order to isolate activity related to spatial attention shifts specifically. General linear model analysis using the constructed learning and attention predictors revealed heightened activation for both tasks in bilateral superior parietal lobules (SPL), bilateral V5, and bilateral middle frontal gyri (MFG). Increased response during learning was seen in the SPL and V5, but not MFG. Repeated measures ANOVA indicated significant interactions between region and task, as well as a right-biased tendency in the hemisphere^*^task interaction. This suggests a role for the SPL and V5 during sequence acquisition that cannot be explained by attention alone.

## Introduction

The encoding of long visuospatial sequences is broadly relevant in natural settings, where the delayed imitation of spatial targets presents a unique form of learning. When navigating a new environment, for example, it is not necessarily possible to incrementally ascertain whether the correct steps are being taken until the final target has been reached. Conscious awareness of the unchanging path (or sequence) results in the recruitment of explicit memory processes both structurally and functionally distinct from those utilized for implicit tasks (Buckner et al., 1995; Seger et al., 2000), which occur without awareness and whose mechanisms in this context have received far greater focus.

Previous studies using delayed imitation paradigms have suggested the existence of two parallel processes for the learning of long visuospatial sequences (Hikosaka et al., 1998, 1999, 2002). The first of these involves the explicit learning of a sequence of sensory events and recruits prefrontal regions of the association cortex as well as the anterior caudate nucleus of the basal ganglia. The second among these parallel processes instead supports the implicit acquisition of motor sequences and involves the supplementary motor area and putamen. Explicit processes are believed to dominate early on in sequence learning and their implicit counterparts playing a role during later stages (Hikosaka et al., 1999). Constructing a learning paradigm specifically involving the explicit processing of a long series of targets without intermediary feedback therefore serves well to elucidate the neuroanatomical correlates of this form of visuospatial sequence acquisition. The general understanding of the functional anatomy believed to be involved during the processing of long visuospatial sequences also includes, in addition to the superior parietal lobules (SPL), the middle frontal gyrus (MFG) (Shen et al., 1999; Vallar et al., 1999), middle occipital gyrus (MOG) (Beyh et al., 2022; Renier et al., 2010), and precuneus (Ghaem et al., 1997; Suchan et al., 2002), among others (Cona and Scarpazza, 2019). As explicit learning relies crucially on the hippocampus for encoding and consolidation of long-term memory (Basu and Siegelbaum, 2015; Kandel et al., 2014, 2021), its activation is therefore also expected during the learning of long visuospatial sequences. However, distinct regions are likely recruited during the attentional selection of the visuospatial information to be transferred to long-term retention. Precise identification of these specific functions goes beyond the scope of this study, but may include attribution of saliency (Itti and Koch, 2001; Töllner et al., 2011), processing of visual input, spatial processing, or pattern recognition (Park et al., 2022; Strasburger et al., 2011).

As objects must naturally be attended to in order to undergo acquisition in an explicit context, a broad functional overlap may exist between brain activity produced by visuospatial sequence learning and activity that occurs while performing a visual attention task without a learning component (LaBar et al., 1999; Zanto et al., 2011). Accordingly, a main focus of our study was to understand the ways in which brain regions relate to the processing (e.g., attentional selection) specifically associated with explicit visuospatial sequence learning.

Furthermore, recent findings by our group have highlighted the relevance of our Delayed Imitation of Long Spatial Sequences (DILSS) paradigm not only for understanding the foundations of sequence learning, but also for clinical practice. For example, performance on this paradigm is able to distinguish between controls and patients with temporal lobe lesions in cases when the standard battery of neuropsychological tests cannot (Eggert et al., 2022). This renders it a promising tool for the more precise assessment of patient outcome following resection in response to temporal lobe epilepsy. Further investigation into the specific regions involved is therefore warranted in order to better provide an anatomical basis for its clinical utility.

Expected regions recruited by visuospatial attentional demands primarily include those comprising a network dedicated to externally controlled visual attention, the Dorsal Attention Network (DAN). The DAN consists of the intraparietal sulcus, the frontal eye fields, middle temporal region/V5, superior parietal lobule, ventral premotor cortex, and inferior and superior pre-central sulci, among others (Corbetta and Shulman, 2002). The right dorsolateral prefrontal cortex may also be involved (Uddin et al., 2019).

To address these common underlying mechanisms, we devised two analogous experiments both involving visuospatial targets. The first paradigm consisted of a learning task and the second, an attentional paradigm not involving learning. In our primary paradigm, we had participants learn and reproduce (by ocular fixation) a repeating target sequence that they were consciously aware would remain identical across all trials. They were asked to reproduce this 20-target long sequence only after its full presentation and without intermediate feedback. Given these characteristics, the paradigm was labeled the “Delayed Imitation of Long Spatial Sequences” (DILSS) and resembled the early explicit learning phase of paradigms used by Hikosaka et al. (1999). Prior work by our group (Drever et al., 2011a) using the DILSS paradigm has provided strong evidence that the nature of its induced learning is indeed explicit, with recent findings demonstrating impaired task performance in subjects with unilateral temporal lobe resections (Eggert et al., 2022). As the temporal lobe, and more specifically hippocampus, is a necessary component for explicit (Scoville and Milner, 1957; Squire and Zola, 1996), but not implicit memory (Gagnon et al., 2004; Squire, 1992), this signals an explicit and potentially episodic mechanism may be at work. An earlier study of ours also revealed the absence of implicit features such as chunking or error propagation (Eggert et al., 2014). This distinction is critical, as explicit awareness in learning is believed to recruit separate cognitive resources from those used in implicit tasks. Previous research has suggested a dissociation in function such that, in addition to activating different regions, concurrent engagement of both implicit and explicit encoding leads to interference between the two (Kantak et al., 2012; Klimkowicz-Mrowiec et al., 2008). It is therefore of great interest to investigate brain activation during visuospatial processing in a clearly defined explicit context.

Our second task was designed with the aim of eliciting attentional mechanisms associated with visuospatial selection but unrelated to learning. It involved subjects only attending to certain targets at certain times in a visual discrimination task without a repeating sequence being present. By performing both paradigms in the fMRI scanner, the present study aimed to compare the brain activity resulting from these analogous tasks and thereby reveal their individual contributions to explicit visuospatial sequence learning.

Due to the aforementioned functional overlap caused by explicit learning's attentional requirements, direct comparison of changes in activation between the two tasks would not be informative on the differences related to the presence of learning. That is to say, when performing an analysis of variance on blood-oxygen-level-dependent (BOLD) response values, a main effect of *task* should be of only minor interest. Similarly, a main effect of regions activated would only serve to confirm differences in function across the brain and not provide useful information beyond that. Accordingly, small differences in the temporal structure of the learning and attention predictors could affect the main effect of factor *task*, but not the second-order interaction between the factors *task* and *region*. This would also apply to differences between the physical properties of each task's visual stimuli. We therefore focus on interaction effects for understanding the contribution of a visuospatial learning component to the patterns in brain activity that may also be produced by attentional mechanisms.

To summarize, the present study addresses the following questions: first, how brain activity during the acquisition of a visuospatial memory sequence differs when comparing a currently-appended target to all other elements of the same sequence, and second, whether brain activity during visuospatial attentional selection depends on whether or not it occurs in the context of sequential memory encoding.

## Materials and methods

### Subjects

A total of 50 subjects were recruited. However, due to selection criteria described below, only 34 subjects (17 female, mean age 27 ± 7, range 19–53 years) were included in the final data analysis. Subjects were selected for participation only after passing a self-reported screener for contraindications to MRI scanning, any psychiatric/neurological conditions, and handedness (right-handers only). All subjects possessed normal or corrected-to-normal vision. Ethical approval was granted by the medical faculty of the Ludwig-Maximilians-Universität München (registration nr. 20-0981). Recruitment took place by word-of-mouth and online advertisements. All participants were fully informed on the experimental procedures and provided written consent in accordance with the Declaration of Helsinki.

### Setup

Our study utilized a Siemens Prisma 3T scanner (Erlangen, Germany) with a 64-channel head coil. Right eye position was acquired using an EyeLink 1000 Plus (SR Research, Ottawa, Canada), running at 1 kHz. Visual stimulation was provided by a rear projection system (PROPixx projection system, VPixx Technologies Inc., Saint-Bruno, Canada) with a resolution of 1,929 × 1,080 pixels and a horizontal projection size of 48.4 cm, running at a frame rate of 120 Hz. The visual target in the learning task was a white cross on a black background (width: 0.4 cm, bar thickness: 0.08 cm). In the attention paradigm, irrelevant targets consisted of two concentric circles with diameters 3.2 and 1.6 cm (line width of 0.16 cm). The discrimination targets were square boxes with a width of 3.2 cm (line width of 0.16 cm). The character “E” or “mirror-E” is printed at the center of the box. The height of the letters is 2.6 cm. The viewing distance was 125.5 cm. All visual stimuli were generated by a custom made software in MATLAB (Mathworks, Natick, USA) using the Psychtoolbox (Brainard, 1997). In order to directly capture eye movements from within the scanner, we attached a mirror system to the head coil. This setup consisted of a surface mirror tilted 45 deg toward the back of the bore, which allowed us to both project screen contents into the subject's field of vision as well as reflect pupil positions toward the MRI-compatible infrared camera of the Eyelink eyetracker located behind the bore.

### Learning paradigm

**Figure 1:**
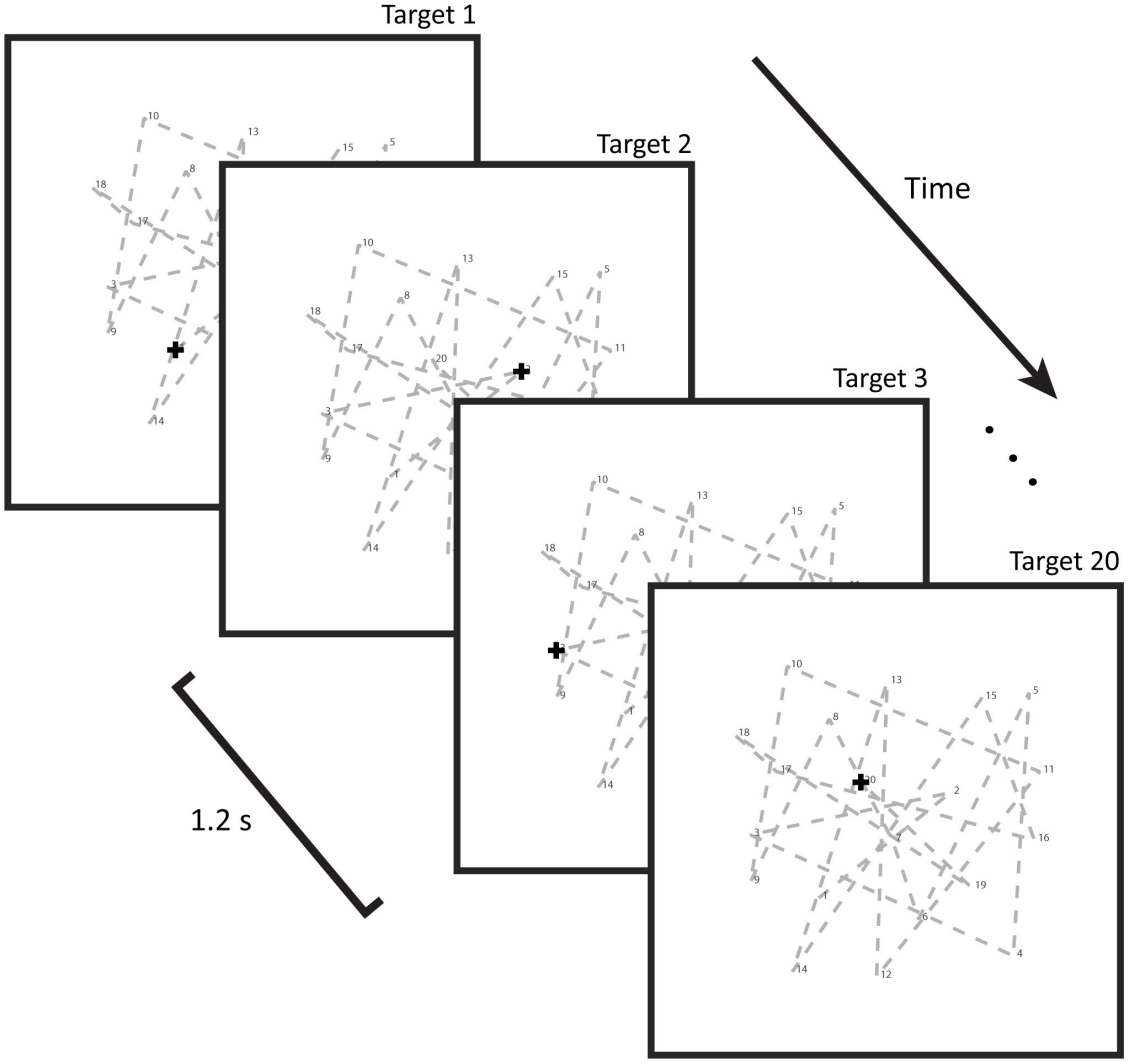
Illustration of the learning paradigm: a cross-shaped target steps every 1.2 s through a predetermined, pseudorandom, 20-target long geometric sequence that remains constant across all 25 trials. Subjects were instructed to fixate on the start position and only observe using peripheral vision, as to remove the influence of oculomotor learning. Following presentation of all 20 targets, the sequence was to be reproduced by eye movement to the locations recalled, as measured by eye tracking. Performance during this retrieval phase was used to retrospectively assess the time points at which particular targets were being learned in the previous presentation phase.

In our deferred imitation of long spatial sequences (DILSS) paradigm, subjects viewed a pseudorandom sequence of 20 two-dimensional targets on a screen, presented at 1.2 s intervals. Targets in this pseudorandom sequence were a minimum of 3.7 cm apart. Within a 5.6 cm radius around each target, only one additional target location was allowed to be present. During presentation, participants were instructed to suppress reflexive saccades and instead remain focused on the initial target position. Fixation was verified by the EyeLink recording. Following consecutive presentation of all 20 targets (plus starting position), subjects were asked to replicate the sequence via visual fixations on the recalled target locations. The classification of fixations as either reproductions or task-irrelevant explorations is described below (Data analysis). Upon reaching the stage in the visual reproduction where they were no longer certain of the next position in the sequence, subjects were instructed to immediately end the trial by button press, rather than guess. This button press would then trigger the presentation of the start position and 20 targets again, with the subjects instructed to first make a saccade to the start position. The sequence presentation started 1.2 s later. For the purpose of establishing a baseline measurement, the target remained at the starting point for 20.2 s every third trial. Presentation and reproduction phases alternated in this manner for a total of 25 trials. The total duration of the learning paradigm was 22.0 ± 3.1 min. An illustration of the paradigm is shown in Figure 1. Breaks of fixation during the presentation phase did not abort the current trial but were analyzed offline.

### Attentional task

**Figure 2:**
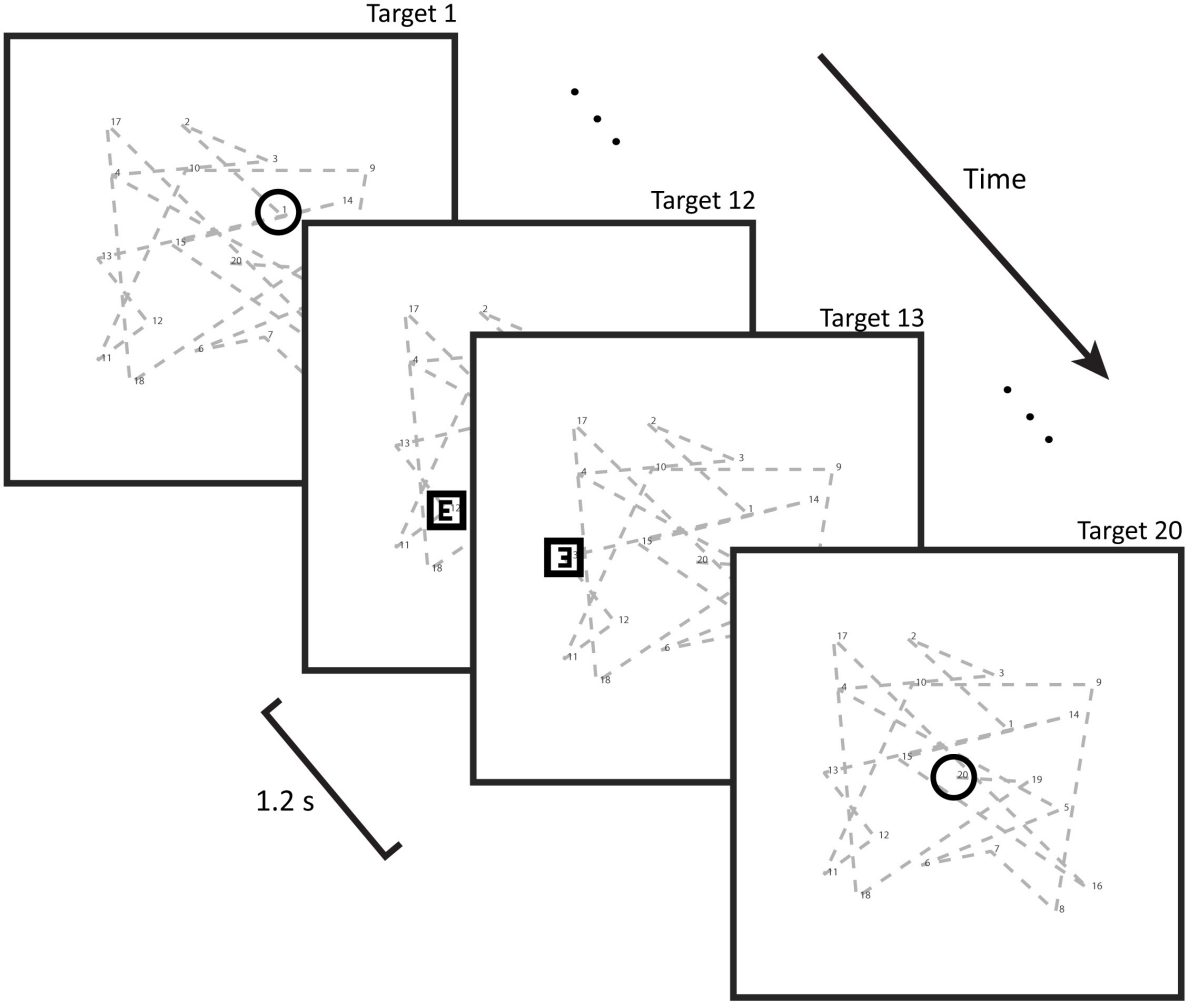
Illustration of the spatial attention task: this task involved selective attention to certain shapes (squares) and not others (circles). Attention was induced by asking subjects to completely disregard the circles and identify the contents of the squares (in the form of either an E or a mirror-E) and report on their quantities by button press. Button responses merely indicated whether there had been an equal or unequal quantity of Es and mirror-Es. Shapes stepped through a pseudorandom non-repeating sequence at the same pace as the targets in the learning task (every 1.2 s). Twenty trials were performed, over which the window of attention (square presentation) gradually shifted toward the end of the trial.

In order to isolate the brain regions specific to spatial processing during learning, we devised a shorter second task intended to only recruit spatial attention, without any learning (Figure 2). This task consisted of 20 trials throughout which the subject's eyes were to remain focused on the center of the screen, using only peripheral vision in order to eliminate oculomotor activity. Each trial featured targets both spatially and temporally distributed in the same manner as those from the learning task (identical number of targets and inter-target interval duration). The sole exception was the fact that the sequence did not repeat every trial but rather was displayed anew over each of the 25 trials, sampled from the same spatial distribution. Task trials consisted of three portions. The first was comprised of three white circles, which were to be disregarded. Following this came a presentation of four white squares to which the subjects should direct covert attention. During this second segment, the squares contained variable symbols, with either an “E” or “mirror-E” shape in the center. The subjects were asked to keep a mental count of how many “E”s vs. “mirror-E”s were present. Finally, another series of white circles appeared which were to be ignored as before. The screen then went black, upon which subjects informed on their mental tally by pressing particular buttons on a button board to indicate either an equal or unequal quantity of both symbols. For example, if two “E”s and two “mirror-E”s were present, the subject would select “equal.” Upon button press, the next trial would initiate, whereby the number of circles in the initial segment increased by one, and the length of the final segment with circles decreased by one. In this way, the temporal aspect of attention and nonattention to targets was analogous to that of the learning task. However, as the spatial sequences differed between all trials, participants did not engage in any learning. While the attention task was specifically designed not to elicit learning, featuring no repeating sequences that could be learned, it might stand to reason that some implicit learning might be taking place with regards to performance. However, subjects were required to delay their response until the end of the trial. An improvement in reaction time, for example, would therefore not be applicable. The induced attentional state was therefore comparable to that of the learning task, despite not relying on learning. The total duration of the attentional task was 9.8 ± 0.4 min. The percentage of correct responses was evaluated.

### Study design

Prior to the scanner session in which the learning and attention tasks were performed, a series of screenings and purely behavioral trainings took place. Subjects were included only after passing a self-reported screener for contraindications to participation, as described above. Spatial working memory was evaluated using the clinically proven Corsi Block test (Berch et al., 1998), in which nine virtual blocks on a monitor lit up in a particular sequence that was to be repeated using the computer mouse. Correct repetitions extended the length of the sequence by one. Upon two incorrect tries, the test ended. Two versions of this test were performed, requiring recall either in the same or reverse order as presentation. The maximum length of correctly recalled sequence defined the forward or backward Corsi span, whose average was used to define the Corsi span. This was followed by a first session of behavioral learning task and a second session of the learning and attentional tasks in the scanner at a later date. As our spatial memory task required a certain degree of focus and saccade repression, a purely behavioral analog of the in-scanner experiment was conducted in advance in order to assess the subjects' ability to successfully complete it, as well as to familiarize the subject with the learning paradigm in a setting where communication could easily take place. Subjects who could not successfully complete the experiment (for example, plateauing in progress after a few targets or quitting the task altogether) after two training sessions were excluded from further participation. In total, we examined 50 participants in the behavioral training sessions, but only 42 of these in the scanner. In-scanner behavioral data was then also used for identifying insufficient learning progress in the second session. Criteria for exclusion consisted of *initial learning speed* being less than zero during the first 10 trials and *final recall probability* being < 0.5. Using these metrics (see section *Behavioral variables*), we retained 34 subjects for the final fMRI analysis.

### Data analysis

#### Eye position calibration

The first step in analyzing behavioral task performance was the calibration of the subject's pupil location (gaze position) to eye position on the projection screen, expressed in cm. A special calibration paradigm was recorded before the beginning of the behavioral tasks, rather than to use the standard EyeLink Calibration performance. Eye position was calibrated using a nine point calibration sequence consisting of four targets at 12 cm eccentricity in the horizontal and vertical direction, four in the corners of a 12 cm × 12 cm square, and one in the center. Each calibration target was presented seven times for 1.2 s. Fixations were included in the calculations when eye velocity remained below 70 deg/s for at least 0.15 s. In each of the nine fixation clusters, fixations with a Mahalanobis distance larger than $\sqrt{-2\;\ln\left(0.02\right)}$ were removed as outliers, corresponding to the 98% confidence interval. Following the removal of outliers, the medians of the fixation clusters replaced the individual fixations as calibration input. A 2D polynomial of the 2nd order was used as a calibration function to map the raw horizontal/vertical eye position ṟ_*x*_/ṟ_*y*_ onto the calibrated horizontal/vertical eye position ${\underline{\text{c}}}_{x}$/${\underline{\text{c}}}_{y}$:


$$\begin{array}{l} \;\left[{\underline{\text{c}}}_{x},{\underline{\text{c}}}_{y}\;\right]=\\ \left[\underline{1},\;{ṟ}_{x},\;{ṟ}_{y},\;{ṟ}_{x}.*{ṟ}_{y},\;{ṟ}_{x}.^{\wedge }2,\;{ṟ}_{y}.^{\wedge }2\right]\;\left[{\underline{\text{p}}}_{x},\;{\underline{\text{p}}}_{y}\right]. \end{array}$$

Each of the vectors ${\underline{\text{c}}}_{x},\;{\underline{\text{c}}}_{y},\;{ṟ}_{x}$, and ṟ_*y*_ in this equation has nine elements, corresponding to the nine fixation positions. The 12 12 parameters (i.e., the two 6D vectors ${\underline{\text{p}}}_{x},\;{\underline{\text{p}}}_{y}$) of this function were by the least square method on the difference between the calibrated cluster centers centers ($\left[{\underline{\text{c}}}_{x},{\underline{\text{c}}}_{y}\;\right]$) and the target positions ([ṯ_*x*_, ṯ_*y*_])


$$\begin{array}{l} \left[{\underline{\text{p}}}_{x},\;{\underline{\text{p}}}_{y}\right]=\text{argmin}||\left[{\underline{\text{c}}}_{x},{\underline{\text{c}}}_{\;y}\;\right]-\left[{ṯ}_{x},{ṯ}_{y}\;\right]|{|}_{F}, \end{array}$$

where ||**A**||_*F*_ denotes the Frobenius norm of the matrix **A**. In a hierarchical backward regression, this full polynomial was compared with a reduced polynomial under exclusion of the mixed term


$$\begin{array}{l} \left[{\underline{\text{c}}}_{x},{\underline{\text{c}}}_{y}\;\right]=\left[\underline{1},\;{ṟ}_{x},\;{ṟ}_{y},\;{ṟ}_{x}.^{\wedge }2,\;{ṟ}_{y}.^{\wedge }2\right]\;\left[{\underline{\text{p}}}_{x},\;{\text{p}}_{y}\right]\;\\ \;\left(10\text{parameters}\right) \end{array}$$

and with a linear calibration function


$$\begin{array}{l} \left[{\underline{\text{c}}}_{x},{\underline{\text{c}}}_{y}\;\right]=\left[\underline{1},\;{ṟ}_{x},\;{ṟ}_{y}\right]\;\left[{\underline{\text{p}}}_{x},\;{\underline{\text{p}}}_{y}\right]\left(6\text{parameters}\right). \end{array}$$

In 9/13/12 of the 34 subjects the calibration resulted in full polynomial, reduced polynomial/linear functions.

#### Applying calibration to task data

As the learning paradigm lasted longer than 20 min, the calibration functions established during the calibration paradigm were modified by a running offset $\left[{\underline{o}}_{x},\;{\underline{o}}_{y}\;\right]$


$$\begin{array}{l} \left[{\underline{\text{c}}}_{x},{\underline{\text{c}}}_{y}\;\right]={\underline{F}}_{\text{c}al}\;\left({\underline{\text{p}}}_{x},\;{\underline{\text{p}}}_{y}\right)+\;\left[{\underline{o}}_{x},\;{\underline{o}}_{y}\right], \end{array}$$

where ${\underline{F}}_{\text{c}al}\;\left({\underline{\text{p}}}_{x},\;{\underline{\text{p}}}_{y}\right)$ denotes the calibration established by the calibration paradigm. The dimension of each of the vectors ${\underline{\text{c}}}_{x},\;{\underline{\text{c}}}_{y},\;{\underline{o}}_{x}$, and ${\underline{o}}_{y}$ is the number of samples recorded during the behavioral task. The running offset was established by a interpolation of grid-points placed at the beginning of every fifth trial. The grid values were fitted to minimize the squared distance between the calibrated fixation locations at the start of each trial and the start location itself. In this way, the calibration corrected for slow head movements during the measurement.

#### Assigning fixations

In order to assign visual fixations to their intended targets, as well as avoid the assignment of unrelated explorations, we applied an algorithm previously developed by our working group (Drever et al., 2011b). This algorithm prioritizes the order of the reproduced sequence over the strict spatial accuracy of its constituent target fixations, while also considering omissions, explorations, and order errors. Through the recursive ordered assignment of fixations, it identifies the longest continuous sub-sequence of targets that can be assigned in order, then removes them from the original sequence and restarts, running until no possible assignments remain.

#### Behavioral variables

The recall probability was defined for each trial as the number of reproduced and assigned targets divided by the sequence length (20). The *initial learning speed* was computed as the regression slope of the recall probability expressed as a function of trial number. The *final recall probability* was defined as the average of the recall probability across the last five trials.

#### Acquisition of imaging data

##### Scan sequences

For the first functional task (learning paradigm), whole brain functional images were collected using echo-planar imaging (EPI) sequence with a voxel size of 2.5 × 2.5 × 2.5 mm and a repetition time of 700 ms (echo time 33 ms, flip angle 45 deg, 54 slices, no-interslice gap, FoV 210 mm, matrix 54 × 84 × 84 px, phase encoding direction A/P, multiband acceleration factor 6). Following this, anatomical T1-weighted MPRAGE imaging was conducted (voxel size 0.75 × 0.75 × 0.75 mm, repetition time 20.6 s, flip angle 12 deg, slice thickness 0.75 mm, 256 slices, FoV 240 mm, matrix 256 × 320 × 320 px, phase encoding direction A/P, GRAPPA acceleration factor PE 2). A subsequent second functional scan with identical scanning parameters was then performed during the attentional component of the experiment.

#### Analysis of fMRI data

##### Preprocessing

MR images received standard preprocessing performed using SPM12 (v7771) in MATLAB (version 2017b) and Penny et al. (2011). Data was corrected for motion by realignment, inspected for artifacts, and coregistered between functional and anatomical scans. Slice-time correction was not applied. Correction for head motion was performed using the “Realign and Unwarp” module in SPM set to default parameters, including registration to the first image volume of the time series. Spatial normalization was obtained by segmentation of the high-resolution T1-weighted images using the segmentation algorithm included in the CAT12 toolbox (version CAT12.7 r1742) as well as the “optimized shooting” spatial registration method likewise included (Ashburner and Friston, 2011). The flow field obtained by this segmentation step was then entered into the “Normalize: Write” SPM module as the deformation field to create versions of the individual functional and structural images in MNI space. Smoothing was applied using an 8 mm FWHM Gaussian kernel.

##### Single subject analysis

The fMRI datasets acquired during the learning and attention tasks were analyzed with their corresponding predictors, which were used as regressors of the BOLD-response in separate general linear models as implemented in SPM (Penny et al., 2011). To eliminate low frequency signal drifts, we applied the SPM-default high-pass filter with a time constant of 128 s. The six head movement parameters provided by SPM (*x, y, z, roll, pitch*, and *yaw*) were entered into the first-level general linear model as nuisance regressors.

**Figure 3:**
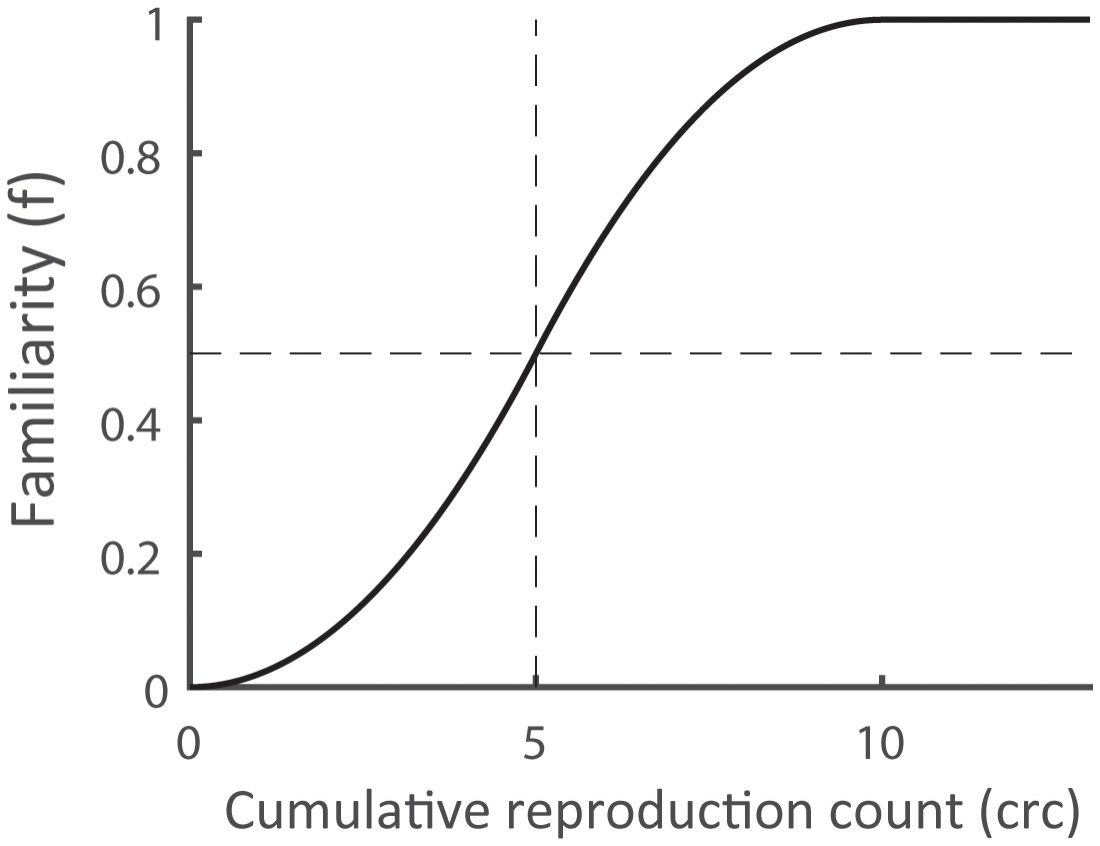
Assumed dependence of the familiarity (f) on the cumulative reproduction count (crc). The function values f = 0/1 indicate that the corresponding target is completely unknown/fully familiar. The threshold value s (= 0.5) defines the count for which the familiarity reaches a value of 0.5.

**Learning predictor:** To isolate the components of the fMRI dataset that corresponded to brain activity during the presentation of targets currently being learned (as opposed to the observation of targets either known or still unfamiliar), we referred to the results of the learning task. That is, behavioral data from the reproduction phase was used for defining the learning predictor, but no predictor was generated to investigate brain activity during the reproduction phase. We computed the learning predictor based on both target familiarity and target novelty. For each trial, we counted the number of times a target had been correctly reproduced in the repetition phase, defining target familiarity (*f* ) as the output of a sigmoid function applied on this cumulative reproduction count (*crc*).


$$f(i,n)=\{\begin{array}{c} \;\frac{1}{2s^{2}}\text{ c}r\text{c}{\left(i,n\right)}^{2}\text{ ​ for }0\leq \text{c}r\text{c}(i,n)<s\\ 1-\frac{1}{2s^{2}}{\left(2s-\text{c}r\text{c}\left(i,n\right)\right)}^{2}\text{ for }s\leq \text{c}r\text{c}\left(i,n\right)<2s\\ \;1\text{ for }2s\leq \text{c}r\text{c} \end{array}\;,$$

where 1 ≤ *i* ≤ 20 denotes the target index and 1 ≤ *n* ≤ 25 the trial number. The sigmoid function (Figure 3) models the learning progress as a gradual increase from an unknown target (*f* = 0) to a familiar target (*f* = 1). The threshold *s* was set to *s* = 5, assuming that a target was fully familiar after it had been successfully reproduced 10 times. This assumption was based on the results of previous studies (Drever et al., 2011a; Eggert et al., 2014, 2022) demonstrating that in DILSS, the length of the memorized sequence successively increases and, once included in this memorized sequence, targets can still be recalled a week later. During training, it was observed that following the first successful target reproduction, its spatial accuracy does not improve much in subsequent trials (Drever et al., 2011b). These results suggest that learning during DILSS represents a successive transfer of spatial working memory content into sequential episodic long-term memory. They further indicate that learning progress in this task is constrained to the few items being learned in a particular trial. This therefor indicates that a crucial component of the learning process is taking place around the time of the presentation (observation) of the target currently being appended to the sequence.

The neuronal activity associated with the degree of learning taking place was modeled by the *learning progress* (*lp*(*i, n*)) predictor, defined as the partial derivative of the *familiarity*


$$\begin{array}{l} l\text{p}\left(i,n\right)=\frac{\delta }{\delta n}f\left(i,\;n\right) \end{array}$$

This definition is consistent with the idea that the brain activity associated with the transfer of a currently presented target into long-term memory depends on its change in familiarity. The above definition of learning progress ensures that only targets with an intermediate degree of familiarity induce learning progress, whereas unfamiliar and fully familiar targets do not. However, this definition is not without alternatives. For example, there is the possibility that the transfer of information regarding target *i* continues during the presentation of target *i*+1, even when target *i*+1 is either unknown or fully familiar. Such a mechanism would correspond to a distribution of the learning progress along the time axis (i.e., along the columns of the predictor matrix (*lp*(*i, n*)). It is important to note that our current approach does not consider such mechanisms. The *learning progress* predictor only extracts learning activity that is strictly linked to the appearance of each target and does not consider activity present during the reproduction phase.

**Attention predictor:** We designed the attention task to elicit spatial attention in an analogous manner to that of learning task. That is, attention was expected to be recruited at time points corresponding to the parts of the learning paradigm presentation phases where the learning progress predictor was expected to show large values. In contrast to the learning task, the attentional discrimination task did not require learning but presumably elicited very similar control of spatial attention. The values of the attention predictor were set to one for the scans sampled during the presentation of the discrimination targets and zero otherwise. As with the learning task, all samples not acquired during the presentation phase were eliminated prior to fitting the data with the general linear model. Thus, brain activity during the latency of the subject responses as well as during the button press did not affect the fitting of the attentional response.

All predictors were expressed as matrices with the target index *(i)* as row index and the trial index *(n)* as column index. Each element corresponded to the time point at the center of target *i*'s presentation interval in the presentation phase of trial *n*. Assuming that during the reproduction phase each target's *familiarity* remained constant and *learning progress* was zero, we extended these prediction matrices into the reproduction phases. The predictors for the entire timespan of the experiment were obtained by concatenating all columns of the extended prediction matrices into a single time sequence, which was then linearly interpolated onto the centers of each fMRI scan. Finally, the predictors were convolved with the hemodynamic response function as provided by SPM (function spm_hrf) and all samples not acquired during the presentation phase were eliminated.

##### Group level

At the group level, we first performed one sample *t*-tests on the single subject contrasts of parameter estimates (beta values) of the learning predictor as well as attention predictor, separately. These parameter estimates are hereafter referred to as learning response or attentional response, respectively. We applied False Discovery Rate (FDR) correction for multiple comparisons to all analyses at voxel level. Results were considered significant at *p* < 0.05.

#### Region of interest determination

Unsurprisingly, since learning requires attention (Craik et al., 1996), there was a near-complete spatial overlap between the learning and attention responses (such that the learning response was contained within the regions of attention task activation). We were interested in only considering the areas common to both experiments. To this end, we created binary masks for both conditions that corresponded to all regions significant at an FDR threshold of 0.05, along with an additional inclusive mask for gray matter. Only the intersection between all three masks with cluster sizes >100 voxels were considered regions of interest. Seven of such regions of overlapping activity were found throughout the parietal, temporal, and frontal lobes. On each side of the parietal cortex were two adjacent clusters which were combined, resulting in a total of five regions of interest, each defined by a corresponding binary mask. We then calculated the center of mass for each region of interest (Table 1). Mean beta values for each ROI were calculated on the subject level.

**Table 1:** Center of mass per region of interest (MNI-coordinates).

***Region of interest***	***x***	***y***	***z***
Left V5	−45	−69	5
Right V5	47	−62	1
Left SPL	−24	−58	55
Right SPL	27	−58	50
Left MFG	−29	0	59
Right MFG	26	4	57
Right MFG (inferior)	43	36	29

Seven regions of interest were established.

##### Comparison of learning and attention responses across different brain areas

To determine whether the activation in both tasks was solely a product of attention, we performed a repeated measures ANOVA on the average individual responses from the regions of interest, with factors *task* (learning, attention), and *region* (left SPL, right SPL, left V5, right V5, left MFG, right MFG, inferior right MFG). That is, the ANOVA was performed using the unstandardized beta coefficients of the regression of the general linear model estimated in each subject. As previously explained in the introduction, a main effect of factor *task* would be of only minor interest, as it cannot directly imply learning specificity. The same would apply for a main effect of the factor *region*. In contrast, a first order interaction effect between factors *task* and *region* could indeed be informative on the relative influence of a learning component on attentional processes in and across our regions of interest. We utilized a multivariate approach to repeated measures ANOVA, since it does not rely on the assumption of sphericity like standard repeated measures ANOVA.

## Results

### Behavioral statistics

**Figure 4:**
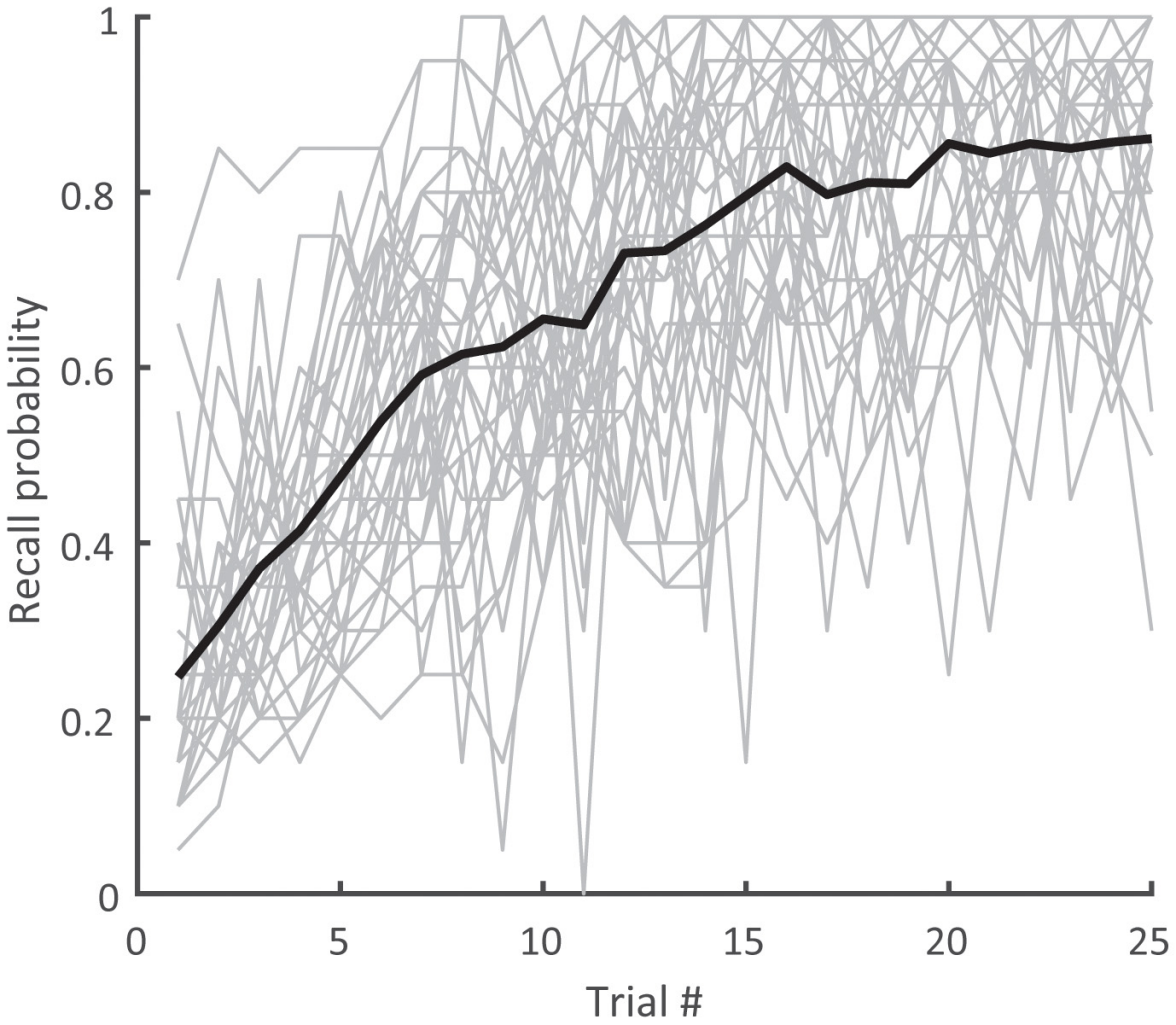
Recall probability over trials for the individual subjects (gray) and their average (black). Recall probability was defined for each trial as the number of reproduced and assigned targets divided by the sequence length (20).

Among the 34 analyzed subjects, the average learning task duration was 22.0 ± 3.1 min and average attention task duration 9.8 ± 0.5 min. All included subjects were able to consistently append targets to the memorized sequence. During the 24 s presentation phase, subjects maintained fixation for the most part. Saccades with amplitudes greater than 2, 5, and 10 deg occurred at a median rate of 8.28 [12.6]%, 1.66 [3.08]%, and 0.18 [0.36]% per subject and per trial. The average distance of the targets from the fixation target was 11.7 ± 3.8 deg. Thus, although fixation interruptions occasionally occurred, the subjects did not follow the targets with their eyes and the targets were presented only in the visual periphery. They initially learned an average of 1.0 new targets per trial (Figure 4). Recall probability, as seen averaged across all subjects in Figure 4, reached its maximum by around trial 15 and subsequently plateaued.

We characterized the behavioral performance by the Corsi span (5.88 ± 1.12 items), the final recall probability (0.85 ± 0.11), and the fraction of exploratory saccades with respect to all saccades in the learning task (0.36 ± 0.15). Corsi span and final recall probability did not correlate with each other [*T*_(32)_ = 0.60; *p* = 0.56]. The percentage of correct responses in the attention task was 95 [15]% [median (interquartile range)].

In order to assess the relationship between these behavioral parameters and the learning response in the selected regions of interest (SPL, V5, MFG), we computed one multiple regression for each parameter (Corsi span, final recall probability, fraction of exploratory saccades) using the learning responses in the respective ROIs as regressors. None of the overall r-squares reached significance (Corsi span: *r*^2^ = 0.26; *F*_(6, 27)_ = 1.62; *p* = 0.18; final recall probability: *r*^2^ = 0.15; *F*_(6, 27)_ = 0.77; *p* = 0.60; fraction of exploratory saccades: *r*^2^ = 0.14; *F*_(6, 27)_ = 0.70; *p* = 0.65). Despite not reaching significance with regard to the learning response, our behavioral results regardless demonstrate that the selected subjects were indeed able to consistently acquire the sequence in the learning task, with recall probability saturating two-thirds through the experiment trials and with minimal saccades.

### Visuospatial sequence learning

**Figure 5:**
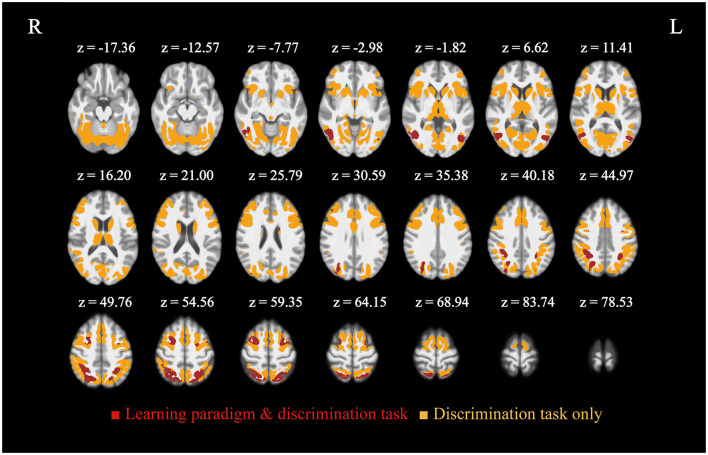
Activation elicited by the learning and attention paradigms in form of multiple comparison corrected T-maps (FDR ≤ 0.05, *n* = 34) displayed over the mean of subject T1-weighted images. Regions common to both learning and attention are shown in red, those exclusive to attention are in orange. Learning paradigm activity was wholly encompassed by that from the discrimination task, no region was observed outside of it. Significant clusters of common activity were found bilaterally, namely in V5 (middle temporal gyri), middle frontal gyri, and superior parietal lobules.

Whole brain analysis of encoding via the generated learning predictor revealed localized activity in parietal, temporal, occipital, and frontal regions (Figure 5). Significant clusters of activity were found in the right middle temporal gyrus (V5), left middle occipital gyrus, bilateral superior parietal lobules, left superior frontal gyrus, left inferior occipital gyrus, and bilateral middle frontal gyri. MNI coordinates and *T-values* are reported in Table 2.

**Table 2:** MNI-coordinates of activations related to the learning and attention predictors (FDR ≤ 0.05, height threshold *T* = 2.93 for learning, *T* = 2.15 for attention).

**Region**	**Learning**			**Attention**			***T***
	***x***	***y***	***z***	***x***	***y***	***z***	
Left superior frontal gyrus	−19	−7	54				3.56
Right inferior temporal gyrus				37	−58	−9	12.18
Right middle temporal gyrus	49	−65	4				10.85
				59	−29	−15	2.67
				55	−30	−15	2.48
Left inferior occipital cortex	−46	−71	8				8.27
	−34	−69	7				3.41
				−41	−70	−9	11.42
Right superior parietal lobule	31	−65	56				6.01
	12	−68	61				5.84
	18	−69	57				5.83
	26	−66	58				5.69
	31	−44	44				5.29
	25	−60	65				5.16
				33	−48	42	12.42
Left superior parietal lobule	−26	−62	64				7.59
				−30	−51	49	11.14
Right anterior insula				32	21	2	14.48
Left anterior insula				−30	21	2	14.41
Right supplementary motor area				6	22	44	12.18
				2	10	56	11.89
Left supplementary motor area				−8	13	48	13.10
Left precentral gyrus	−45	2	54				3.36
				−42	3	30	11.50
Right middle frontal gyrus	43	37	29				3.26
				44	9	34	10.71
				41	30	28	10.41
Left middle frontal gyrus	−30	1	64				4.63
	−40	1	59				3.44
Right angular gyrus				30	−66	34	10.79
Left supramarginal gyrus				−39	−44	42	10.49
Right orbitofrontal cortex				21	34	−15	2.70
				19	30	−17	2.41
Left orbitofrontal cortex				−21	42	−13	2.29
Right middle cingulate gyrus				7	−28	28	2.44

### Visuospatial attention

Our second task elicited broad activation consistent with the Dorsal Attention Network (Corbetta and Shulman, 2002; Fox et al., 2005), with the resultant regions entirely encompassing those found for the visuospatial sequence learning task. Regions of significant activity included the left anterior insula, right middle temporal gyrus, bilateral superior parietal lobules, bilateral precentral gyri, right angular gyrus, bilateral middle frontal gyri, left supramarginal gyrus, right middle cingulate gyrus, bilateral inferior temporal gyri, and orbitofrontal cortex, along with additional motor regions. MNI coordinates and *T-values* are reported alongside those for learning in Table 2.

**Figure 6:**
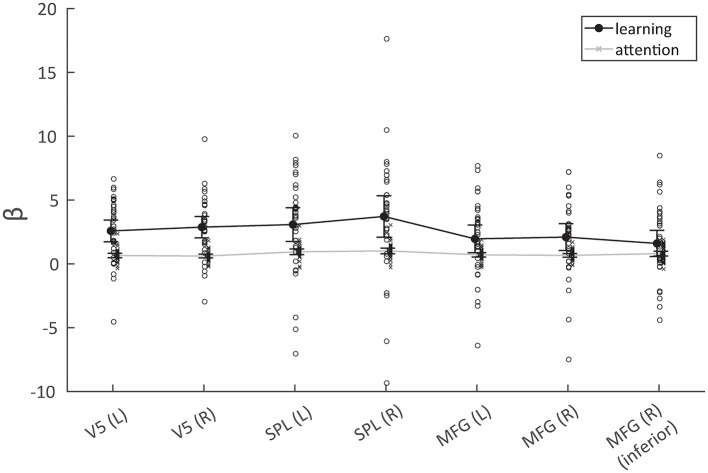
Response in terms of parameter estimates for the seven analyzed regions across the two experiments (learning/attention). The BOLD response was significantly higher for the learning condition in V5 and the superior parietal lobules (SPL), but less so in the middle frontal gyri.

A repeated measures ANOVA with factors *task* (learning/attention) and *region* [V5 (L), V5 (R), SPL (L), SPL (R), MFG (L), MFG (R), and MFG (R, inferior)] revealed a significant interaction effect between *task* and *region* [*F*_(5, 165)_ = 4.00, *p* = 1.87^*^10^−3^]. This interaction (Figure 6; Table 3) was due to there being greater differences in activation between tasks for the SPL and V5 than for the MFG. A further analysis featuring factors *task, region*, and *hemisphere* revealed a tendency toward right-biased asymmetry across all regions [task^*^hemisphere: *F*_(1, 33)_ = 3.68; *p* = 6.38^*^10^−2^], though this was not significant for the interaction between *task, region*, and *hemisphere* [task^*^region^*^hemisphere: *F*_(2, 66)_ = 0.54; *p* = 0.58]. In the ANOVA with only factors *task* and *region*, there were also significant main effects of both factors [task: *F*_(1, 33)_ = 16.68, *p* = 2.65^*^10^−5^; region: *F*_(5, 165)_ = 7.05, *p* = 5.00^*^10^−6^]. However, as explained in the introduction, these are of minor interest.

**Table 3:** Results of multivariate ANOVA with factors *task* (learning/attention) and *region* of observed activity [seven regions: bilateral V5/middle temporal areas, bilateral superior parietal lobules, and bilateral middle frontal gyri (+1)].

**Effect**	***F***	***p***
Task: *F_(1, 33)_*	16.681	0.000265
Region: *_*F*(5, 165)_*	7.0530	0.000005
Task^*^Region: *_*F*(5, 165)_*	4.0037	0.001870

Significant interactions were found for task and region, as well as main effects of task and region, though these were of only minor interest.

## Discussion

### Summary of results

Our study revealed regional activation associated with the explicit acquisition of long visuospatial sequences, along with the activity recruited by non-learning-based visuospatial attention. In the learning condition, activity was found bilaterally in V5 and the superior parietal lobules, as well as in the bilateral middle frontal gyri/frontal eye fields. The purely attentional spatial task elicited broader activity consistent with the Dorsal Attention Network (Corbetta and Shulman, 2002). When comparing average response across regions of interest from the learning vs. attention results, increased activation was observed for V5 and SPL but less so for the MFG. Curiously, neither working memory span nor final sequence recall probability correlated significantly with the learning response in any of the ROI. The fraction of exploratory saccades was likewise uncorrelated with the learning response.

### BOLD activation from learning paradigm

#### V5

Prominent activation of V5 by the spatial learning task serves to confirm its role in the processing of moving visual stimuli (Maunsell and Van Essen, 1983; Moutoussis and Zeki, 2008). Interestingly, however, although this function is considered by some (Wilson et al., 1992) to also be attributable to V1, we did not find activity here when using our learning predictor. This suggests that there may indeed be a specific role performed by this occipito-temporal region during the learning of visual sequences (Fan et al., 2020). For example, it has been suggested that separate pathways exist for the processing of slow vs. fast visual motion, wherein V1 is bypassed in the case of fast movements. In this case, subcortical structures are thought to convey visual information directly from the superior colliculus to V5. A study by Grasso et al. (2018) demonstrated that when V5 activity was transiently disrupted by transcranial magnetic stimulation, subjects performed more poorly on a spatial processing task involving fast (23 deg/sec motion) but not slow (4.4 deg/sec) stimuli. However, this direct colliculo-extrastriate pathway is primarily believed to support implicit processing (Grasso et al., 2018), whereas our paradigm involved explicit awareness of the learning task.

Perhaps unsurprisingly, V1 was not found to be more heavily recruited when targets were in the process of being learned, when contrasting with those either already committed to long-term memory or far ahead enough in the sequence to not yet be attempted. One explanation would be that V1 activity was similarly taking place across the entire experiment, further indicating that it is not being specifically recruited during the processing of targets that are being learned. We did also observe stronger response in V5 than in V1 in the attention task.

#### Middle frontal gyrus/frontal eye field

Both conditions elicited activity bilaterally in the middle frontal gyrus. The left precentral gyrus was also present in both tasks. As the frontal eye fields are known for their role in directing top-down, goal-directed allocation of visual attention, this is consistent with literature (Crowne, 1983; Cazzoli et al., 2015; Veniero et al., 2021). Mechanisms are believed to involve direct mediation of excitability of lower level visual areas through perceptually-relevant shifts in neural oscillations (Veniero et al., 2021). Additionally, this region also plays an established role in mental rotation/imagery in spatial contexts (Cona and Scarpazza, 2019). However, due to its explicit recruitment during attentional orienting, this function likely contributed to a relatively diminished difference in activation when comparing the learning to attention tasks, further suggesting this region was not specific to learning.

#### Superior parietal lobule

The contribution of the superior parietal lobule (SPL) to visuospatial processing and memory has been demonstrated by numerous studies. A meta-analysis of hundreds of imaging studies on spatial cognition further consolidated the functions of the superior parietal lobule across related cognitive domains, particularly spatial attention, visuospatial working memory, and spatial imagery (Berthoz, 1997; Tagaris et al., 1998; Cona and Scarpazza, 2019). In our study, the superior parietal lobule was bilaterally activated in both the learning and attention tasks, but with greater activation in the learning condition. Of interest is its purported role in spatial imagery, which would be necessary during explicit recall of a visual sequence. As, by definition, this function should be similarly activated in explicit encoding, it may be the case that this parietal activity supports a top-down mechanism reflective of progress in the learning of the sequence.

Applied in a broader context, the SPL has also been shown to be involved during reading, as processing text also requires the ordered incorporation of visual targets into a larger series. This can be illustrated through the phenomenon of simultanagnosia. In this neurological disorder, patients experience an extreme restriction of visuospatial attention, leaving them able to only attend to one item at a time (Wolpert, 1924). The functional components of this condition can be deduced through the presence of lesions in the superior parietal lobule (SPL) (Vialatte et al., 2021), a region where hypoactivity has also been observed in subjects with developmental dyslexia (Peyrin et al., 2011; Reilhac et al., 2013). Its activation during the acquisition of less contextual sequences in our paradigm would consequently also be expected.

#### Lateralization

In terms of hemispheric differences, literature has indicated a lateralization of function with regards to both working and long-term memory specialization, with the right hemisphere being more heavily recruited during spatial tasks (Smith and Milner, 1981; van Asselen et al., 2006) and the left hemisphere preferentially activating during verbal and object-based memory exercises (Dennis and Whitaker, 1976; Ojemann and Dodrill, 1985). Our results corroborate these conclusions, in that a slight, right-biased asymmetry between hemispheres was present in the activation patterns we observed.

#### Absence of hippocampus

The hippocampus is not only encoding at the moment of presentation, but likely also engaged in the continued consolidation of earlier targets. Research has indicated that long-term consolidation can indeed begin immediately following encoding, as per analysis of changes in default mode network complexity (McDonough et al., 2019). It would therefore be reasonable to assume sustained hippocampal activation throughout the experiment, which could result in a lack of significant activation when contrasting presumed encoding of a specific target with all other activity.

### Influence of saccade suppression

The attention paradigm we employed was derived from one used by Schneider and Deubel (1995) to test the effect of saccade planning on the attention shifts that subserve peripheral discrimination prior to saccade execution. For context, it is understood that there is a lowering of detection thresholds at target locations when covert attention is shifted toward the target location (Cameron et al., 2002). Unlike in the Schneider and Deubel (1995) study, however, our paradigm involved the suppression of saccades. Instead, subjects fixated on the center of the screen during all stages of the task to eliminate oculomotor activity. However, despite this intention, covert attention shifts can also occur for microsaccades, as seen in a study investigating microsaccade directionality within a delayed saccade task (Laubrock et al., 2005). Despite saccade suppression, a shift in attention toward discrimination targets did take place in our attention paradigm, as suggested by the high percentage of correct responses. In the original study Schneider and Deubel (1995), subjects responded correctly for < 80% of the unattended discrimination targets, whereas it was 95% in our study. The observed activation patterns in our attention task were highly consistent with known visuospatial attention network components, assuring that attention shifts took place in spite of the instructed suppression.

### Activation from attention paradigm

#### Dorsal attention network

The regions activated by our attentional task largely reflected the components of the dorsal frontoparietal network (Corbetta and Shulman, 2002; Fox et al., 2005; Szczepanski and Saalmann, 2013) including the superior parietal lobule and frontal eye fields. Given that our attention paradigm was explicitly designed to elicit top-down attention to the visuospatial targets, this is as expected (Herbet and Duffau, 2022). Furthermore, when comparing the average activity of our ROIs, we found greater differences between conditions for the superior parietal lobule and V5 than for the MFG (indicated by the significant interaction between *region* and *task*). This lends support to the notion that the frontal regions we observed reflect attentional mechanisms not involved with the learning of visuospatial sequences.

#### Parietal and temporal responses as marker for explicit visuospatial processing

When comparing learning task performance (final recall probability, specifically) with beta values in regions of interest as generated using our learning predictor, we did not find a significant correlation. This suggests that the responses we observed in the parietal and temporal ROI, although they were specific to the learning task, were not themselves predictive of the quantity of stored information, nor how efficiently it was stored, but rather marked brain activity related to a learning-specific processing of this visuospatial information.

#### Evidence for explicitness

On the question of whether our learning task was truly explicit in nature, the extensive overlap between learning and attention-only conditions lends support to that conclusion, as implicit learning would not have required attention and a dissociation in functional neuroanatomy would have more likely been present (Vuilleumier et al., 2005). For example, an early fMRI study on implicit visual memory found increased activation in the bilateral lingual gyri in response to the learning of deliberately ignored items; this is distinct from the regions we observed. Furthermore, a Wilkinson et al. (2010) study using transcranial magnetic stimulation found that inhibiting the primary motor cortex abolished implicit learning, showing it to be critical to this form of memory. Activation stemming from our learning paradigm did not include M1, lending additional support to the notion that we may have indeed initiated explicit memory mechanisms. Furthermore, previous findings by our group (Drever et al., 2011a) have shown that fixation accuracy in the DILSS task does not improve across subsequent trials, indicating the absence of implicit learning. That is, the slope of the linear regression of the reproduction error on trial number did not differ significantly from zero, while the mean of this slope across subjects was negative (−0.01 deg/trial). Compared to the error present in the first reproduction (around 2.5 deg), the total improvement in accuracy after 25 trials was only 10%. This absence of improvement therefore indicates that implicit learning plays a subordinate role in this paradigm, even if it cannot be completely ruled out. Likewise, we cannot from our findings conclude that the activated regions play no role in implicit learning. However, the embedding of learning-specific neuronal activity in attention-specific activity in our experiment suggests that both paradigms rely on similar mechanisms, which are likely explicit.

#### Sensitivity to encoding

It is important to address the functional overlap in regions identified by the learning predictor, in that they may also be attention-related. However, by contrasting encoding vs. non-encoding states, we were able to identify those that, while also involved with attention, are additionally sensitive to encoding. The SPL (particularly the precuneus), for example, is sensitive to item familiarity (Kim and Cabeza, 2007; Wheeler and Buckner, 2003) and V5 is known to feature priming-sensitive cells (DeAngelis and Newsome, 2004), alongside its role in attention orienting. We therefore do not consider this as confounding, but rather an affirmation of the current understanding of the roles played by V5 and the SPL.

#### Applications

As previously mentioned, there exist potential clinical applications for our findings. Previous research by our group (Eggert et al., 2022) on the encoding of long visuospatial sequences in temporal lobe resection patients demonstrated the usefulness of the DILSS paradigm in detecting impairments in visuospatial memory. Unlike other currently used measures of memory, such as the California Verbal Learning (Elwood, 1995) or Rey-Osterrieth Complex Figure Tests (Shin et al., 2006) (whose application did not indicate memory impairments in the patient sample), the DILSS paradigm was indeed able to differentiate between patients with unilateral temporal lobe resection and healthy controls. The present study contributes a complementary perspective on these prior findings, offering neurological insights into the potential mechanisms utilized by the brain during the performance of our task. For example, the right-biased hemispheric asymmetry in BOLD activation associated with sequence acquisition validates the previous study's finding of poorer performance when subjects had undergone resections in the right temporal lobe.

#### Limitations

Unfortunately, due to the demanding nature of the experiment, eight out of 42 scanned subjects were unable to adequately learn the sequence and were therefore ultimately excluded from final analysis. This may have introduced a bias toward faster-learning subjects who found the task easier and possibly acquired the sequence using different neurocognitive resources. As an example, some subjects may have relied more heavily on their superior working memory capacity, rather than encode one target per trial, as was the average rate. This could be resolved in future studies by implementing more stringent screening during the initial behavioral training prior to scanning.

Furthermore, differences between our two tasks carry the possibility of introducing nuisance variables into our analysis. There was, however, a significant interaction effect that indicated the parietal and temporal regions of interest were more responsive to the presence of learning than the MFG was. This cannot plausibly be attributed solely to factors such as target shape or other visual context. For example, targets moved in a similar fashion and although target shape differed, it would be reasonable to assume that our parietal activation was more related to object localization than shape identification. It is also less plausible that the activity in V5 in response to sequence learning demands would be a consequence of such differences in shape. Despite these task differences, their overall contexts were kept as similar as possible. The visual environment was consistent, with white targets against a black background. The distance and angle of target motion during presentation was also similar. Most importantly, the timing of attentional focus was deliberately designed to be analogous, as described in the Methods section. Moreover, a prior study (Gauthier et al., 2002) that also made use of a task involving discrimination between mirrored objects specifically did not find activity in the SPL, as we did. Differences in target shape and character complexity are therefore unlikely to account for the increased activity in the SPL and V5 during the DILSS paradigm. Rather, the specificity of this activation is more likely due to the presence of learning, reflecting associated processes underlying it.

An additional limitation lies in the methodology itself, with 3T fMRI potentially being insufficient for detecting small regions with a low signal-to-noise ratio. Significant activity in the superior colliculus, for example, was not observed. However, its location in the midbrain and proximity to vasculature as well as other sources of physiological noise may have masked its signal. This could potentially be mitigated through the use of a 7T scanner.

## Conclusions

Our study aimed to identify the brain regions associated with the processing underlying the acquisition of long visuospatial sequences, utilizing both the DILSS learning paradigm and an analogous spatial attention-only task. Results indicated that V5 as well as parietal (but not frontal) regions show a learning-specific enhancement of attention-related activity. Our findings thereby corroborate past literature on their role during visuospatial processing (Cona and Scarpazza, 2019; Maunsell and Van Essen, 1983), within the context of sequence learning. They furthermore substantiate past research on spatial attention (Corbetta and Shulman, 2002) and hemispheric asymmetry during visuospatial processing (Jacobs et al., 2010), providing valuable insight into the potential mechanisms through which learning is made possible.

## Data Availability

The raw data supporting the conclusions of this article will be made available by the authors, without undue reservation.
